# Dynasore - not just a dynamin inhibitor

**DOI:** 10.1186/s12964-015-0102-1

**Published:** 2015-04-10

**Authors:** Giulio Preta, James G Cronin, I Martin Sheldon

**Affiliations:** Institute of Life Science, College of Medicine, Swansea University, Swansea, SA2 8PP UK

**Keywords:** Dynasore, GTPase, Dynamin, Endocytosis, Cholesterol, Lipid rafts

## Abstract

Dynamin is a GTPase protein that is essential for membrane fission during clathrin-mediated endocytosis in eukaryotic cells. Dynasore is a GTPase inhibitor that rapidly and reversibly inhibits dynamin activity, which prevents endocytosis. However, comparison between cells treated with dynasore and RNA interference of genes encoding dynamin, reveals evidence that dynasore reduces labile cholesterol in the plasma membrane, and disrupts lipid raft organization, in a dynamin-independent manner. To explore the role of dynamin it is important to use multiple dynamin inhibitors, alongside the use of dynamin mutants and RNA interference targeting genes encoding dynamin. On the other hand, dynasore provides an interesting tool to explore the regulation of cholesterol in plasma membranes.

## Introduction

**Figure 1:**
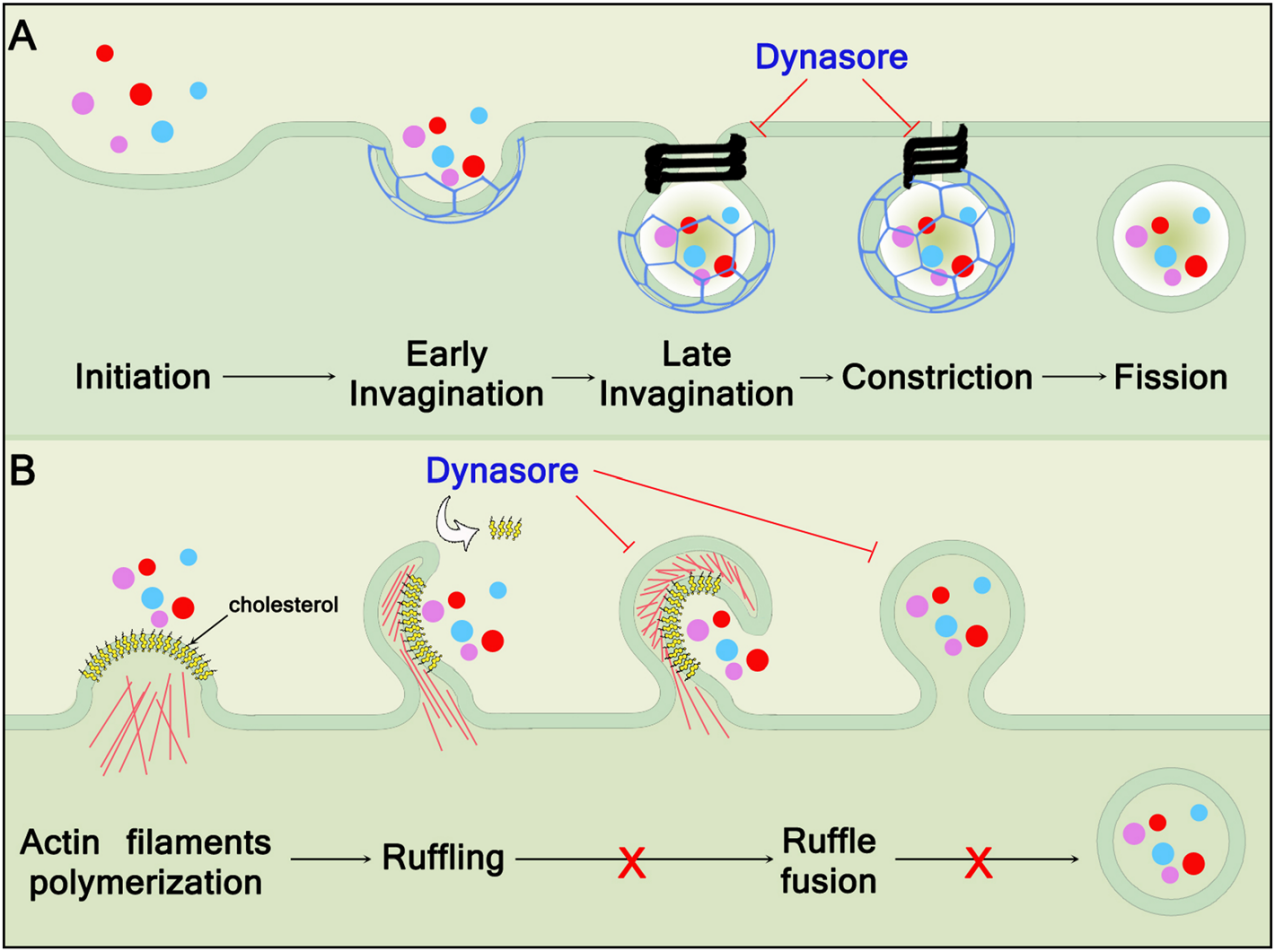
**The stages of clathrin-coated vesicle formation. (A)** Inititation and early invagination: a clathrin-coated pit is formed and cargo-specific adaptors are selected. Late invagination: further clathrin is recruited and polymerizes in hexagons and pentagons to form the clathrin coat. Constriction: dynamin is recruited to the neck of the forming vesicle where it forms helical structures, and induces membrane scission. Fission: an endocytic vesicle is produced containing cargo molecules. Dynasore inhibits the GTPase activity of dynamin, blocking constriction and fission. **(B)** The steps leading to macropinocytosis. During the vesicular trafficking process, cellular membranes undergo dynamic morphological changes, in particular at the vesicle generation and fusion steps. Macropinocytosis involves the eruption of membrane ruffles from the cell surface that can fuse with the plasma membrane to engulf surrounding cargo, a process that requires extensive actin mobilization. Macropinosomes then fuse with compartments of the normal endocytic pathway. Dynasore reduces plasma membrane cholesterol, inhibiting mobilization of the cellular membrane.

Dynamin is an intracellular protein with essential roles in membrane remodelling and fission of clathrin-coated vesicles formed during endocytosis, and vesicles that bud from the trans-Golgi network [1]. In particular, endocytosis is dependent on dynamin for the invagination of plasma membrane to form clathrin-coated pits, and dynamin polymerizes to form a helix around the neck of budding vesicles of plasma membrane leading to membrane fission and generation of free clathrin-coated vesicles (Figure 1A) [2]. Clathrin-mediated endocytosis regulates fundamental cellular processes, including the homeostasis of plasma membrane, receptor turnover, and the uptake of nutrients [3]. On the other hand, many pathogens have evolved to exploit endocytosis to enter eukaryotic cells. As well as linking to the actin cytoskeleton during clathrin-coated vesicle formation, interaction between dynamin and the actin cytoskeleton occurs during the formation of membrane ruffles, lamellapodia, and podosomes [4-6]. In addition, a growing number of dynamin-like proteins have been identified, such as mitochondrial DRP1, which contribute to the fusion and remodelling of intracellular membranes [1,7].

Dynamin is a 100 kDa protein with multiple domains, principally explored by generation of dynamin mutants. Perhaps the most important domain is a large GTPase enzyme essential for membrane fission [8,9,10]. In addition to the GTPase domain, dynamin also contains a pleckstrin homology domain implicated in membrane binding, a GTPase effector domain essential for self-assembly, and a C-terminal proline-rich domain, which contains several SH3-binding sites [1]. Dynamin partners bind to the proline-rich domain, stimulating dynamin’s GTPase activity and targeting dynamin to the plasma membrane [11]. In particular, dynamin is efficiently supplied with GTP by interaction between the dynamin proline-rich domain and nucleoside diphosphate kinases NM23-H1/H2, to trigger membrane fission [12]. Purified dynamin exists as a tetramer [13], which can self-assemble into structures that resemble rings and helices [14]. In *Drosophila melanogaster* and *Caenorhabditis elegans* only one dynamin isoform has been identified [15-17]. However, three dynamin-encoding mammalian genes (*DNM1*, *DNM2* and *DNM3*) have been identified [18,19]. Although the dynamin isoforms have similar functions, including membrane fission during clathrin-mediated endocytosis, dynamin 1 and dynamin 3 are mainly expressed in the brain, whereas dynamin 2 is expressed ubiquitously [1]. Although overexpression of mutants has been used to explore the role of dynamin, even mutations that effectively target the dynamin GTPase, such as dynamin K44A, S45N, T65F and T65A, vary in their potency and the stage at which they inhibit endocytosis [8,20]. Whilst the overexpression of dynamin mutants and RNA interference targeting the mammalian dynamin genes has been valuable, progress in understanding the mechanism of action of dynamin has also benefited from the discovery of dynamin inhibitors, including the GTPase inhibitor dynasore [2].

### Evolution of dynamin inhibitors

**Figure 2:**
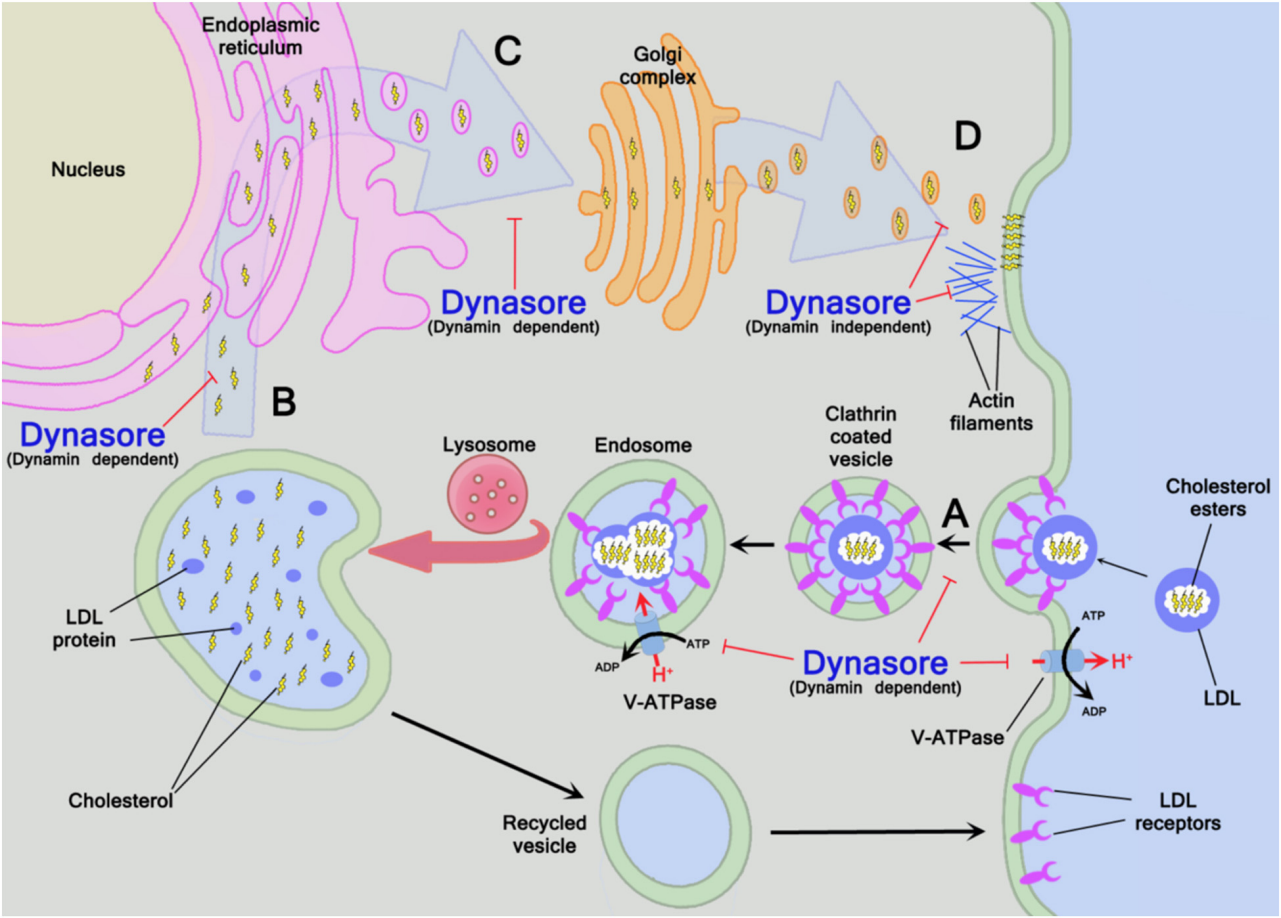
**Dynamin-dependent and dynamin-independent effects of dynasore. (A)** Dynasore inhibits the GTPase of dynamin, which prevents clathrin-coated endocytosis, including internalisation of LDL receptors in the plasma membrane and inhibits the vesicular H^+^-ATPase, which is involved in cholesterol recycling from endosomes back to the plasma membrane. **(B)** Dynasore also inhibits the movement of LDL-derived cholesterol from the endolysosomal network to the endoplasmic reticulum. **(C)** In addition, dynasore impacts cholesterol homeostasis in a dynamin-dependent manner, decreasing the amount of cholesterol in the Golgi apparatus. **(D)** Dynasore, perhaps by remodelling actin filaments, as well as reducing plasma membrane cholesterol, disperses the organization of lipids in lipid rafts.

The first dynamin inhibitors to be identified were ammonium salts, such as myristyl trimethyl ammonium bromides (also known as MiTMAB), and the dimeric tyrphostins [21,22]. Most of the first generation of dynamin inhibitors, and their subsequent derivatives, prevent recruitment of dynamin to membranes. On the other hand, compounds that inhibit ATPases and GTPases, for example dynole 34–2 or dynasore inhibit the activity of dynamin following recruitment of dynamin to plasma membranes [23]. Dynasore was identified by Macia and colleagues by screening ~16,000 compounds for the ability to inhibit the GTPase activity of dynamin 1, and evidence for the activity of dynasore included inhibition of endocytosis of the transferrin receptor and low density lipoprotein receptor (LDLR) [2]. A characteristic of dynasore is the non-competitive inhibition of the basal and stimulated rates of GTP hydrolysis, without affecting the affinity for GTP binding or dynamin self-assembly [2]. Within 2 minutes, treatment of cells with dynasore inhibits clathrin-mediated endocytosis, and this effect can be reversed in approximately 20 minutes by removal of the inhibitor (Figure 2A and Table 1) [2,24].

**Table 1:** **Evidence supporting dynamin-dependent and dynamin-independent effects of dynasore**

**Dynamin-dependent effects**		
**Effect**	**Supporting approaches**	**Reference**
Clathrin-mediated endocytosis	Dynasore	[1,2,8,20,34]
	Dynamin mutants	
	Triple dynamin knock out	
	Dyngo-4a	
	Dynamin inhibitor peptide	
	siRNA	
Accumulation of cholesterol in ER	Dynasore	[31,32]
	Dynamin mutants	
	siRNA	
Golgi vesiculation	Dynasore	[33,53]
	Dynamin mutants	
	siRNA	
Inhibition of V-ATPase activity	Dynasore	[37]
	Dynamin inhibitor peptide	
	siRNA	
**Dynamin-independent effects**		
**Effect**	**Supporting approaches**	**Reference**
Disruption of lipid rafts	Dynasore differs from dynamin inhibitor peptide and siRNA	[24]
Inhibition of membrane ruffling	Dynasore and Dyngo 4a differ from triple dynamin knock out	[34]
Destabilization of F-actin	Dynasore and Dyngo 4a differ from triple dynamin knock out	[34]

Thus, the discovery of dynasore provided an effective tool to study endocytosis in a range of cell types, and in cells derived from several species, including humans, mice and cattle. However, dynasore also has undesirable properties including the binding of serum proteins, causing the loss of dynamin inhibitory activity [25]. Furthermore, dynasore binds to detergents that are often used for *in vitro* drug screening, which reduces the potency of the inhibitor. These limitations of dynasore lead to the synthesis of dihydroxyl and trihydroxyl dynasore analogs, called the Dyngo compounds, which have improved potency, reduced cytotoxicity, and reduced detergent binding [26].

### Dynasore in the regulation of cholesterol homeostasis: beyond dynamin inhibition

An emerging role of dynamin is the regulation of cellular cholesterol, and dynasore impacts cholesterol homeostasis. Sixty to 90% of cellular cholesterol is located in the plasma membrane, and cholesterol forms about half of the total plasma membrane lipids [27]. Recent evidence supports a concept for three pools of cholesterol in plasma membranes [28]: a labile pool of cholesterol that is depleted when cells are deprived of cholesterol; cholesterol that is bound to sphyngomyelin and is not labile; and, finally an essential pool of cholesterol that is necessary for cell viability. The amount of cholesterol in the labile, sphyngomyelin-bound, and essential pools may vary between types of cells but is around 16%, 15% and 12% of the plasma membranes of fibroblasts, respectively [28].

Cellular cholesterol homeostasis depends on the balance between sequestration of cholesterol in membranes or cholesterol metabolism, and the uptake of LDL-derived cholesterol via endocytosis of the LDLR or cholesterol synthesis via the mevalonate pathway [29]. The LDL-derived cholesterol esters are de-esterified in endolysosomes to release free cholesterol, which transits to the plasma membrane to resupply the pool of labile cholesterol and, once the plasma membrane cholesterol is replete, free cholesterol moves to the endoplasmic reticulum (ER) [28]. Cholesterol synthesis via the mevalonate pathway is controlled by SREBP-2 [30]. When cells have sufficient ER cholesterol, usually > 5% of ER lipids, SREBP-2 in complex with the escort protein (Scap) is bound to an ER membrane anchor protein Insig. However, when ER cholesterol is < 5% of ER lipids, the SREBP-2/Scap complex is released from the ER and transported to the Golgi in COPII-coated vesicles. In the Golgi, SREBP-2 is cleaved to release the active form, which enters the nucleus and drives transcription of genes encoding most components of the mevalonate pathway, and for the LDLR. When there is excess cholesterol, or loss of sphingomyelin, plasma membrane cholesterol is delivered to the ER where it is esterified by the ER resident protein ACAT, and cholesterol esters are stored in cytoplasmic droplets. Dynamin also plays a role in cholesterol homeostasis as LDLR internalization depends on endocytosis [2]. Presumably by inhibition of dynamin-dependent endocytosis, dynasore reduces LDL uptake in HeLA cells to 10% of that of the control [31]. The implication of this observation is that dynasore treatment would then lead to depletion of labile cholesterol in the plasma membrane. However, dynamin also appears to have an additional role in the delivery of free cholesterol from the endolysosomal network to the ER since the use of the K44A mutated form of dynamin, RNA interference targeting dynamin, or dynasore, leads to accumulation of free cholesterol and LDL-derived cholesterol within the late endolysosomal compartment (Figure 2B and Table 1) [31,32]. The importance of dynamin in cholesterol homeostasis is further illustrated by the use of the dynamin K44A mutant, as well as transient transfections with dominant negative mutant constructs of dynamin 1 and dynamin 2, which inhibited cholesterol-induced vesiculation of the Golgi (Figure 2C and Table 1) [33]. Taking the above findings together, one inference is that cells deficient in dynamin or treated with dynasore would not initiate mechanisms to increase cellular cholesterol when cholesterol is depleted in different compartments, because the ER contains surplus free cholesterol. In support of this concept, treatment of cells with dynasore reduces LDLR gene expression, although less rapidly than supplying cells with LDL [31].

As well as effects on cellular cholesterol attributable to inhibition of dynamin, recent observations imply that dynasore also influences cholesterol homeostasis in a dynamin-independent manner. As expected, endocytosis of the transferrin receptor was blocked in fibroblast cells that have a triple knockout of *DYN1*, *DYN2* and *DYN3*, although uptake of dextran, called fluid-phase endocytosis, was not affected [34]. Surprisingly, treatment of the triple knockout fibroblasts with dynasore or Dyngo-4a inhibited fluid-phase endocytosis, implicating dynamin-independent effects of the inhibitors [34]. Furthermore, membrane ruffling was prevented by dynasore or Dyngo-4a but not the triple knockout of dynamin. Membrane ruffles are actin-rich protrusions of the plasma membrane that can be observed on the surface of many cell types, often involved in macropinocytosis (Figure 1B). Macropinocytosis, unlike clathrin-mediated endocytosis, is a dynamin-independent processes [35]. Thus, it appears that dynasore and Dyngo-4a have unexpected off-target effects. One possibility is an effect on plasma membrane cholesterol because extraction of cholesterol with methyl-β-cyclodextrin also inhibits the formation of membrane ruffles at the plasma membrane, and inhibits the reorganization of filamentous actin at the cell periphery necessary for the formation of membrane ruffles (Figure 2D and Table 1) [36]. A further potential mechanism underlying the dynamin-independent effect of dynasore on cellular cholesterol is related to dynasore inhibition of vacuolar H^+^-ATPase (V-ATPase) enzymes (Figure 2D and Table 1) [37]. Inhibition of V-ATPase perturbs clathrin-coated vesicle formation, with retention of cholesterol in non-acidified endosomes, and loss of cholesterol from the plasma membrane, and the effect is partially rescued be providing exogenous cholesterol [38]. Reduction of passive cholesterol efflux from HeLA cells and macrophages also provides supporting evidence that dynasore reduces the labile pool of plasma membrane cholesterol [31]. The mechanism for this “off-target” effect is not known, but it is interesting to note that GTPase activity is important for assembly of the COPII-coated vesicles of liposomes and endoplasmic reticulum [39,40].

### Dynasore targets lipid rafts

Lipid rafts are membrane microdomains that are enriched in cholesterol, sphingomyelin, sphingolipids and phospholipids, and these areas of membrane differ in composition from the surrounding regions of plasma membrane [41,42]. Lipid rafts contribute to the compartmentalization of membranes and the spatiotemporal regulation of cellular signalling. Pathogenic bacteria and viruses also exploit lipid rafts to cause pathology or to gain entry into mammalian cells [43-45]. Microbes not only target the clusters of receptors often concentrated in lipid rafts but also utilize their cholesterol-rich microdomains [46]. In particular, the pore-forming, cholesterol-dependent cytolysins, such as Aerolysin, bind to lipid rafts [47]. Some cholesterol-dependent cytolysins bind to cellular receptors that are enriched in lipid rafts; glycosyl phosphatidylinositol-anchored receptors in the case of Aerolysin. However, other cholesterol-dependent cytolysins, such as Perfringolysin O, bind the labile cholesterol in cellular membranes [28]. Cholesterol-dependent cytolysins multimerise in plasma membranes to form pores, leading to osmotic cell death. As might be expected, depletion of cellular cholesterol using methyl-β-cyclodextrin is protective against the effect of cholesterol-dependent cytolysins [48]. However, dynasore was recently reported to protect HeLA cells and fibroblasts from the toxic effect of two members of the cholesterol-dependent cytolysin family, Pyolysin and Streptolysin-O, as efficiently as methyl-β-cyclodextrin [24]. Moreover, there was evidence that the effect of dynasore was dynamin-independent because RNA interference targeting to reduce dynamin expression did not protect against Pyolysin. The dynamin-independent effect of dynasore was not only associated with reduced cellular cholesterol but also dispersal of plasma membrane lipid rafts (Figure 2D and Table 1). Similarly, the lipid raft-dependent uptake of the subtilase cytotoxin of *Escherichia coli* was suppressed by dynasore, but was not influenced by RNA interference targeting dynamin expression [49]. The importance of disruption of lipid rafts is also supported by studies of innate immunity, where cell plasma membrane receptors such as Toll-like receptor 4 (TLR4) and CD14, which bind the pathogen-associated molecule lipopolysaccharide (LPS), are localised to lipid rafts [50]. Indeed, dynasore also reduced the inflammatory cytokine response to LPS in fibroblasts [24]. It would be interesting for future work to explore if dynasore impacts not only labile cholesterol in plasma membranes, but also the sphyngomyelin-bound cholesterol in lipid rafts. This would be particularly important as statin and cyclodextrin molecules only appear to deplete the labile pool of cholesterol in plasma membranes [28].

In addition to changes in plasma membrane cholesterol, the physical properties of cell membranes and the shape of cells may be modulated by interactions between dynasore and actin. Indeed, dynasore destabilizes and remodels F-actin *in vitro* [51,52]. Dynamin triple knockout cells changed shape following dynasore treatment, providing evidence for a dynamin-independent effects on actin (Figure 2D and Table 1) [34]. Taken together, the inhibition of membrane ruffles and prevention of CDC-mediated cytolysis by dynasore [31,24], implies that dynasore actively influences the content and distribution of cholesterol in plasma membranes, and that this is independent of dynamin. Further work is now need to determine the mechanism by which dynasore exerts dynamin-independent effects on mammalian cells.

## Conclusion

Dynasore provides rapid and reversible inhibition of dynamin-dependent endocytosis, which is effective in cells from several species. However, in addition to inhibition of the GTPase of dynamin, dynasore has wider effects on cellular cholesterol, lipid rafts, and actin. The mechanisms associated with these “off-target” effects require further exploration. Understanding how dynasore modulates plasma membrane cholesterol is particularly intriguing as this may uncover novel methods to counter pathogen entry, and reduce the impact of cholesterol-dependent cytolysins and other pore-forming toxins on cell viability. However, caution is required when using dynasore to determine the role of dynamin in the biology of cells. Robust evidence for the impact of dynamin likely requires the combined use of several dynamin inhibitors, alongside RNA interference targeting the genes encoding dynamin.
